# Improved Extracorporeal Cardiopulmonary Resuscitation (ECPR) Outcomes are Associated with a Restrictive Patient Selection Algorithm

**DOI:** 10.3390/jcm13020497

**Published:** 2024-01-16

**Authors:** Benjamin Assouline, Nathalie Mentha, Hannah Wozniak, Viviane Donner, Carole Looyens, Laurent Suppan, Robert Larribau, Carlo Banfi, Karim Bendjelid, Raphaël Giraud

**Affiliations:** 1 Intensive Care Unit, Geneva University Hospitals, 1205 Geneva, Switzerland; benjamin.assouline@hcuge.ch (B.A.); nathalie.mentha@hcuge.ch (N.M.); hannah.wozniak@hcuge.ch (H.W.); viviane.donner@hcuge.ch (V.D.); carole.looyens@hcuge.ch (C.L.); karim.bendjelid@hcuge.ch (K.B.); 2 Faculty of Medicine, University of Geneva, 1205 Geneva, Switzerland; laurent.suppan@hcuge.ch (L.S.); robert.larribau@hcuge.ch (R.L.); 3 Geneva Hemodynamic Research Group, Faculty of Medicine, University of Geneva, 1211 Geneva, Switzerland; carlo.banfi@unige.ch; 4 Emergency Department, Geneva University Hospitals, 1205 Geneva, Switzerland

**Keywords:** out-of-hospital cardiac arrest, VA-ECMO, ECPR

## Abstract

Introduction: Out-of-hospital cardiac arrest (OHCA) is a leading cause of mortality. Despite decades of intensive research and several technological advancements, survival rates remain low. The integration of extracorporeal cardiopulmonary resuscitation (ECPR) has been recognized as a promising approach in refractory OHCA. However, evidence from recent randomized controlled trials yielded contradictory results, and the criteria for selecting eligible patients are still a subject of debate. Methods: This study is a retrospective analysis of refractory OHCA patients treated with ECPR. All adult patients who received ECPR, according to the hospital algorithm, from 2013 to 2021 were included. Two different algorithms were used during this period. A “permissive” algorithm was used from 2013 to mid-2016. Subsequently, a revised algorithm, more “restrictive”, based on international guidelines, was implemented from mid-2016 to 2021. Key differences between the two algorithms included reducing the no-flow time from less than three minutes to zero minutes (implying that the cardiac arrests must occur in the presence of a witness with immediate CPR initiation), reducing low-flow duration from 100 to 60 min, and lowering the age limit from 65 to 55 years. The aim of this study is to compare these two algorithms (permissive (1) and restrictive (2)) to determine if the use of a restrictive algorithm was associated with higher survival rates. Results: A total of 48 patients were included in this study, with 23 treated under Algorithm 1 and 25 under Algorithm 2. A significant difference in survival rate was observed in favor of the restrictive algorithm (9% vs. 68%, *p* < 0.05). Moreover, significant differences emerged between algorithms regarding the no-flow time (0 (0–5) vs. 0 (0–0) minutes, *p* < 0.05). Survivors had a significantly shorter no-flow and low-flow time (0 (0–0) vs. 0 (0–3) minutes, *p* < 0.01 and 40 (31–53) vs. 60 (45–80) minutes, *p* < 0.05), respectively. Conclusion: The present study emphasizes that a stricter selection of OHCA patients improves survival rates in ECPR.

## 1. Introduction

Out-of-hospital cardiac arrest (OHCA) is a leading cause of mortality, with an annual incidence of 50 to 100 per 100,000 in the general population in Europe and North America [1]. Over the years, Cardiopulmonary resuscitation (CPR) has been improved, and different methods have been studied to increase survival. Nonetheless, the overall survival rate remains low, at 10.7% [2]. Notably, technological progress has been made since the implementation of conventional manual cardiopulmonary resuscitation (CCPR). This includes early cardiac defibrillation and mechanical chest compression devices. Among the new techniques, extracorporeal membrane oxygenation (ECMO) has emerged as a sophisticated alternative to CCPR, thus offering improved blood flow with better organ perfusion and bringing patients closer to appropriate treatment [3]. As ECMO remains an invasive and costly technique, it has been mainly studied in cases of refractory CA and restricted to highly selected subgroups of patients. Various criteria for patient selection are under investigation to establish an algorithm that can predict favorable outcomes with ECPR. One prominent criterion is the time to ECMO implementation, determined by the no-flow and low-flow time. While studies have reported better results with this technique in in-hospital cardiac arrest (IHCA) compared to OHCA, the survival advantage disappears when corrected for the low-flow time [4,5].

In the present clinical investigation, the authors used the institutional ECMO data-base, to assess the impact of implementing a new and more restrictive eligibility algorithm for refractory OHCA patients treated with ECPR. The aim of this study is to compare the two algorithms (permissive and restrictive) and to evaluate the impact of the restrictive algorithm on survival rates with cerebral performance categories (CPCs) scale ≤ 2.

## 2. Methods

### 2.1. Setting

The Geneva University Hospital (HUG) is a tertiary care center located in the western part of Switzerland. It is the regional referral center for mechanical circulatory support and operates as an emergency medical system, offering ECPR to a population of 500,000 residents.

### 2.2. Study Design

This study is a retrospective analysis of a prospective database of patients treated with ECPR for refractory OHCA. All adult patients who received ECPR at HUG between 2013 and 2021 were included. Approval for this study was obtained from the Cantonal Research Ethics Commission of the Republic and Canton of Geneva (2023-00951, 26 July 2023).

### 2.3. Study Protocol

**Figure 1:**
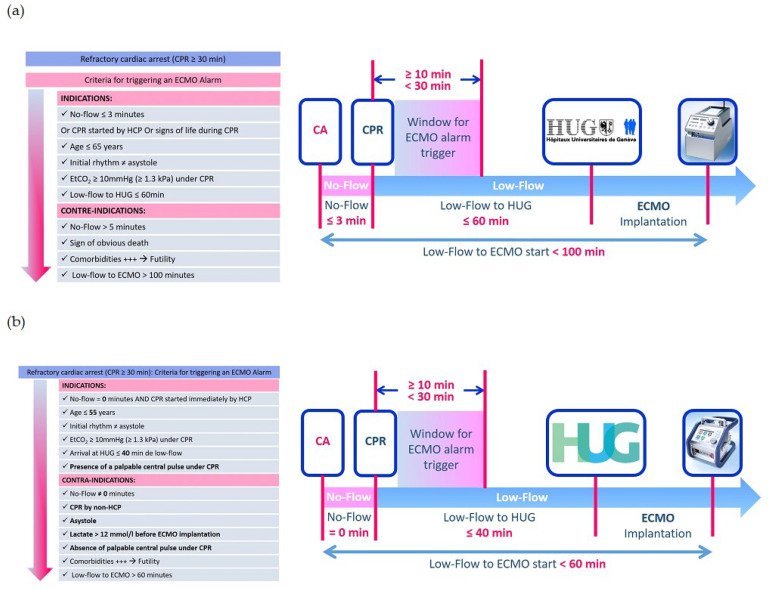
(**a**). Permissive algorithm. CPR: cardiopulmonary resuscitation, ECMO: extracorporeal membrane oxygenation, EtCO_2_: end-tidal carbon dioxide. (**b**). Restrictive algorithm. CPR: cardiopulmonary resuscitation, ECMO: extracorporeal membrane oxygenation, EtCO_2_: end-tidal carbon dioxide, HCP: health care provider.

During this period, two different algorithms (Figure 1) were used. The permissive algorithm was utilized from 2013 until mid-2016. Subsequently, a revised algorithm was implemented based on international guidelines [6,7,8] and our institution’s three-year experience. The revision resulted in a stricter selection of patients facing refractory OHCA. Key differences between the two algorithms include reducing the no-flow time from less than 3 min to 0 min (implying that the cardiac arrest must occur in the presence of a witness with immediate CPR initiation), reducing low-flow duration from 100 to 60 min, and lowering the age limit from 65 to 55 years.

Data were prospectively collected from the ECMO database. Data including age, gender, initial heart rhythm, cardiac arrest cause, no-flow and low-flow times, CPR providers, location of ECMO implantation, pre-ECMO end-tidal CO_2_, pH and lactate, ECMO duration, and cardiovascular risk factors were analyzed. Survivors were defined as ECPR patients alive at 30 days with a cerebral performance category (CPC) score of ≤2.

The aim of this study is to compare historical and new algorithms (permissive (1) and restrictive (2)) to determine if the adoption of a restrictive algorithm was associated with higher survival rates.

### 2.4. Statistical Analysis

Patients were stratified according to the algorithm followed, and a descriptive analysis of patients’ characteristics on admission, time under ECMO, and 30-day survival was performed. Subsequently, patients were stratified according to survival, and the same variables were described. Continuous variables were presented as the median and interquartile range (IQR). Categorical variables are expressed as the number of patients (*n*) and percentage (%). Fisher’s exact test was used to detect differences in categorical variables, and the Mann–Whitney U test for continuous variables. Kaplan–Meier curves were plotted to describe the time under ECMO to death, and a log-rank test was performed to compare the two algorithms. All reported *p* values are two-sided, and statistical significance was defined as *p* < 0.05. Analyses were performed using Stata^®^ IC 16.0 (StataCorp, College Station, TX, USA) and GraphPad Prism^®^ 6 for Windows (GraphPad Software, San Diego, CA, USA).

## 3. Results

Between April 2013 and December 2021, a total of 48 patients underwent ECPR at Geneva University Hospital. A total of 23 patients were considered eligible according to Algorithm 1 and 25 according to Algorithm 2. Throughout the study period, local advanced life support (ALS) included the use of a mechanical chest compression assist device (LUCAS^TM^ 2). The baseline characteristics of the two groups are summarized in Table 1. The main cause of OHCA in the two groups was acute coronary syndrome, accounting for 56.5% in Algorithm 1 and 56% in Algorithm 2. The two groups showed similar characteristics regarding sex, age, and cardiovascular risk factors (overweight, diabetes, dyslipidemia, and hypertension). Algorithm 2 showed a significant difference in survival rate (9% vs. 68%, *p* < 0.05). Moreover, significant differences emerged between the two algorithms regarding the no-flow (0 (0–5) vs. 0 (0–0) minutes, *p* < 0.05), CPR provider (professional in 52% vs. 76%, *p* = 0.03), and the pre-ECMO lactate levels (13.9 (12–15) vs. 9.3 (7–13.3) mmol/L, *p* = 0.02).

Table 2 details the characteristics and differences between survivors and non-survivors. Among the survivors, 17 (89.5%) belong to Algorithm 2 vs. 2 (10.5%) from Algorithm 1. Compared to non-survivors, survivors had a significantly shorter no-flow and low-flow time (0 (0–0) vs. 0 (0–3) minutes, *p* < 0.01 and 40 (31–53) vs. 60 (45–80) minutes, *p* < 0.05), respectively. In addition, survivors had significantly lower pre-ECMO lactate levels (8.6 (6.7–12.1) vs. 13.9 (10.8–15) mmol/L, *p* < 0.05) and longer time on ECMO (96 (48–288) vs. 24 (20–96) hours, *p* < 0.05). Moreover, survivors had significantly fewer cardiovascular risk factors (11 (57.9%) vs. 26 (89.7%), *p* < 0.05). Finally, no significant difference was found between the two groups regarding the initial heart rhythm. It is important to note that four patients presenting asystole received ECPR in both algorithms (10.5% of the cohort).

**Figure 2:**
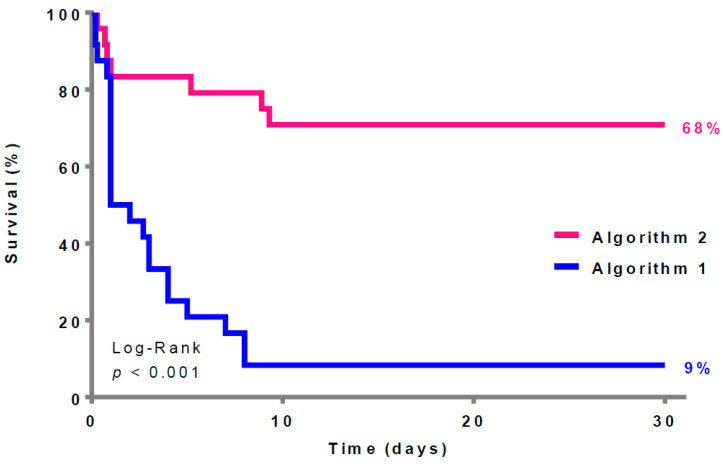
Thirty-day survival according to the two different algorithms (survivors = 19 patients).

The Kaplan–Meier analysis (Figure 2) reveals a significant difference in the survival rates between the two algorithms (Algorithm 1: 2 (9%) vs. Algorithm 2: 17 (68%), *p* = 0.001).

## 4. Discussion

The present retrospective study emphasizes that a stricter selection of OHCA patients improves survival in ECPR. Shorter no-flow and low-flow times, which were the main differences between the algorithms, were associated with significantly higher survival rates. Moreover, as expected, lower pre-ECMO lactate levels were also associated with better outcomes.

Currently, there are no unanimously accepted indications for ECPR. In addition, evidence supporting this costly and resource-intensive technique is lacking. To date, three recent randomized controlled trials (ARREST trial [9], Prague OHCA study [10], and INCEPTION trial [11]) investigated the impact of ECPR on survival in the context of OHCA and yielded contradictory results. The ARREST trial included 30 adult patients (18–75 years) presenting OHCA and refractory VF with no ROSC after three shocks. It was prematurely stopped for ethical concerns. In fact, the authors reported an impressive survival rate of 43% in the ECPR group, compared to 7% in the CCPR group (*p* = 0.023) [9]. The Prague OHCA study randomized 256 adult patients (18–65 years), presenting OHCA with no ROSC after 5 min of advanced life support (ALS), between an invasive strategy and CCPR. The invasive strategy consisted of the following bundle of care: intra-arrest transport with mechanical CPR, ECPR, and immediate invasive assessment with coronary angiography. The trial was stopped for futility. The study was negative and showed a 6-month survival with a cerebral performance category (CPC) (1–2) of 31% in the invasive strategy group and 22% in the control group (*p* = 0.09) [10]. Finally, the INCEPTION trial, the only multi-center RCT conducted, included 134 adult patients (18–70 years), presenting OHCA and ventricular arrhythmia with no ROSC after 15 min of ALS. The study was negative and showed a 30-day survival with a cerebral performance category (CPC) (1–2) of 20% in the ECPR group and 16% in the control group (*p* = 0.518) [11]. While the results of these three RCTs appeared to diverge, they remained interesting. Indeed, after a careful and critical appraisal, several factors seem to explain these contradictory results. Important lessons could be learned from these trials, which will be discussed below.

Several observational studies comparing ECPR with historical case controls investigated factors affecting the outcomes of ECPR [12,13,14]. Among these factors, the no-flow time, corresponding to a period without CPR, appeared to be an important prognostic factor. Indeed, in a retrospective North American study of nearly 7300 OHCA patients, the authors showed that the increase in no-flow time was inversely related to a favorable neurological outcome. The probability of a favorable outcome decreased by 13% for each additional minute of no-flow time [15]. In the present study, Algorithm 2 resulted in a selection of patients with a shorter no-flow duration and was associated with higher survival rates compared to Algorithm 1. Notably, although the timing duration seems to strongly correlate with the overall prognosis, it is essential to emphasize the importance of the reliability of this information when considering ECPR. In fact, to increase the reliability of this timing and in comparison to Algorithm 1, patients were only considered eligible for ECPR if the arrest was witnessed by a professional (physician, nurse, or paramedic) and CPR was initiated immediately. Although this was difficult to measure, it may have impacted the survival rate differences observed in our cohort. It is important to note that although considered a contraindication, six patients (24%) in Algorithm 2 received ECPR because non-HCPs were considered by the emergency medical team (EMT) to have performed effective CPR.

Similarly, the low-flow time is also an important criterion for identifying eligible ECPR candidates, as excessive low-flow times are consistently associated with worse outcomes. Low-flow time for a patient corresponds to the time spent under external cardiac massage without ROSC. While the quality of this resuscitation remains of utmost importance, the duration of this period is seemingly just as important [16]. Indeed, in a Japanese study of nearly 10,000 cardiac arrest patients treated with ECPR, shorter low-flow times were associated with improved survival [17]. This was also observed in RCTs. In fact, the mean low-flow time before VA-ECMO implantation in the INCEPTION trial was 74 min (SD = 18.4), which was longer than initially planned (<60 min) [11]. This is one of the factors that could have impacted the results of this negative study. Another factor that could potentially explain the longer low-flow time observed in this trial resides in the expertise of the ECMO team and center. Indeed, the INCEPTION trial, which included a non-ECLS expert center, reported the longest median time between hospital arrival and the start of cannulation (16 min) and the longest median cannulation duration (20 min) [11]. In comparison, the median total ‘door-to-ECMO time’ was 12 min in the Prague OHCA study, and the mean time from randomization to ECMO initiation was 12 min in the ARREST trial [9]. Both trials were conducted in expert ECLS centers. Several lessons could be learned from these RCTs and should be emphasized. To implement a successful ECPR program for OHCA, the entire resuscitation chain, from the pre-hospital to intra-hospital teams, should aim at the minimization of the no-flow and low-flow times. The decision to proceed to ECPR should be taken as rapidly as possible and should follow strict criteria. Cannulation should be performed by an expert team, dedicated and highly trained, to minimize the time to implantation and to bridge the patient to further care.

Another important marker of CPR quality is the pre-ECMO lactate levels. Lactate is a marker of tissue hypoxia and/or hypoperfusion with anaerobic metabolism. It can be correlated with no-flow and low-flow times [18] and may, therefore, have an impact on the outcomes following a cardiac arrest [19]. Our study found that non-survivors had higher pre-ECMO lactate levels compared to survivors. This is in line with the results of a previous study on 340 OHCA patients, where a serum lactate level < 9 mmol/L was positively associated with patient survival to hospital discharge (OR 2.00, 95% CI 1.01–4.06) [20,21]. All these results are consistent with our findings. Indeed, the main modifications between the two algorithms related to the duration of the no-flow, reduced from <5 min to 0 min, and the low-flow to implantation, reduced from 100 to 60 min. However, our results did not show a significant difference in survival rate according to the presenting rhythm.

In a French cohort of 85 OHCA patients treated with ECPR, the authors compared their data after the modification of their ECPR algorithm, as the new algorithm excluded non-shockable rhythms. After analysis, a sustained shockable rhythm was the only independent predictor of survival to hospital discharge with a good neurologic outcome [22]. In a systematic review and meta-analysis of 841 OHCAs receiving ECPR, the authors showed that an initial shockable rhythm was associated with twice the odds of a favorable neurological outcome than non-shockable rhythms (OR, 2.20; 95% CI, 1.30–3.72) [23]. Interestingly, the Prague OHCA study was the only RCT that included patients with non-shockable rhythms (39% of the enrolled population). In fact, 22% presented asystole as the presenting rhythm. This may have decreased the benefit of ECPR and could potentially explain the non-statistical difference regarding survival. This was emphasized in a post hoc analysis of this trial. Authors reported that the primary outcome was achieved in 40% of patients with a shockable rhythm and only in 5% of patients with a non-shockable rhythm (*p* < 0.001) [10]. This was confirmed recently through an individual patient data-pooled analysis of the ARREST and PRAGUE OHCA trials [9,11]. These results underline that a patient presenting with non-shockable rhythm, especially asystole, may not benefit from this invasive technique and could be excluded from future guidelines regarding OHCA-ECPR. It is to be noted, and contrary to other international ECPR algorithms for OHCA, that patients presenting asystole as the initial rhythm were considered ineligible in our center, both in Algorithms 1 and 2. However, in total, four patients with asystole received ECPR (10.5% of the cohort). This emphasizes that, despite strict guidelines, experienced ECLS physicians frequently deviate from protocol in real life.

The present study has limitations. Firstly, this is a single-center retrospective study that introduces known biases. Furthermore, the conclusions of this study may not be extrapolated to other institutions with a different organization of the care chain and ECPR implementation. Secondly, the study has a limited sample size, which affects its statistical power and generalizability. In fact, a multivariate analysis could not be performed with such a small sample size. Therefore, the authors cannot exclude a selection bias despite a pre-specified research protocol conducted by experienced physicians. Thirdly, the two algorithms followed each other and are thus not contemporary. This raises the risk that the study results may be biased due to the evolution of medical practices and the valuable experience gained by the ECPR team over time. However, the medical management of ECPR patients did not change between the study periods at our institution, particularly regarding target temperature management (TTM), and there were no differences in canulation procedures. Finally, Algorithm 2 resulted in improved survival rates by restricting the inclusion of patients. It remains possible that some patients who could have survived with ECPR were excluded due to the current stricter criteria. This raises ethical concerns, and future RCTs should focus on finding the optimal balance regarding ECPR candidacy.

## 5. Conclusions

This retrospective study on refractory OHCA patients treated with ECPR demonstrates that adopting a more restrictive algorithm, with shorter no-flow and low-flow times, is associated with improved survival. Therefore, these criteria seem essential for the selection of OHCA patients when considering ECPR.

## Figures and Tables

**Table 1:** Comparison of baseline characteristics in the study population between the two algorithms: 2-sided Fischer’s exact test was used for categorical variables, and Mann–Whitney for continuous variables.

	Permissive Algorithm (1) (*n* = 23)	Restrictive Algorithm (2) (*n* = 25)	*p*
Survival, *n* (%)	2 (9%)	17 (68%)	<0.01
First rhythm, *n* (%)			0.43
PEA	5 (21.7)	10 (40)	0.2
VF	15 (65.2)	12 (48)	0.3
VT	0	2 (8)	0.5
Asystole	3 (13)	1 (4)	0.4
Cause of cardiac arrest, *n* (%)			0.8
Coronary artery disease	13 (56.5)	14 (56)	0.6
Pulmonary embolism	3 (13)	4 (16)	1
Rhythm disorder	4 (17.4)	2 (8)	0.4
Other	3 (13)	5 (20)	0.7
No-flow time (minutes), median (IQR)	0 (0–5)	0 (0–0)	<0.01
Low-flow time (minutes), median (IQR)	50 (35–90)	48 (40–60)	0.4
Cardiac massage providers, *n* (%)			0.03
Non-HCP *	11 (47.8)	6 (24)	0.1
Paramedics	9 (39.1)	6 (24)	0.3
Nurse	0	4 (16)	0.1
Doctor	3 (13%)	9 (36)	0.1
Place of ECMO implantation, *n* (%)			0.06
Operating room	9 (39.1)	1 (4)	<0.01
Cath Lab	7 (30.4)	9 (36)	0.8
Emergency room	4 (17.4)	13 (52)	0.02
ICU	3 (13)	2 (8)	0.7
EtCO_2_ before ECMO (mmHg), median (IQR)	30 (14–46)	25 (19.5–35)	0.4
pH before ECMO, median (IQR)	7.0 (6.9–7.04)	7.07 (6.9–7.2)	0.09
Lactate (mmol/L) before ECMO, median (IQR)	13.9 (12–15)	9.3 (7–13.3)	0.02
Time on ECMO (hours), median (IQR)	48 (24–96)	72 (39–213)	0.2
Age (years), median (IQR)	51 (47–60)	56 (51–62)	0.5
Gender, male, *n* (%)	17 (73.9)	17 (68)	0.2
Cardiovascular risk factor overall, *n* (%)	18 (78.3)	19 (76)	0.6
Overweight, *n* (%)	13 (56.)	14 (56)	1
Hypertension, *n* (%)	10 (43.5)	7 (28)	0.3
Hypercholesterolemia, *n* (%)	8 (34.8)	9 (36)	0.4
Diabetes, *n* (%)	5 (21.7)	5 (20)	1
Smoker, *n* (%)	8 (34.8)	7 (28)	0.8

PEA: pulseless activity, VF: ventricular fibrillation, VT: ventricular tachycardia, HCP: health care provider, ECMO: extracorporeal membrane oxygenation, EtCO_2_: end-tidal carbon dioxide, ICU: intensive care unit. * The presenting rhythm, in case of non-HCP initiated CPR was collected from the automatic external defibrillator.

**Table 2:** Comparison between survivors and non-survivors: 2-sided Fischer’s exact test was used for categorical variables, and Mann–Whitney for continuous variable.

	Survivor (*n* = 19)	Non Survivor (*n* = 29)	*p*
Restrictive algorithm, *n* (%)	17 (89.5)	8 (27.6)	<0.01
First rhythm, *n* (%)			0.4
PEA	7 (36.8)	8 (27.6)	0.5
VF	11 (57.9)	16 (55.2)	1
VT	1 (5.3)	1 (3.5)	1
Asystole	0	4 (13.8)	0.1
Cause of cardiac arrest, *n* (%)			0.6
Coronary artery disease	10 (52.6)	17 (58.6)	0.8
Pulmonary embolism	4 (21.1)	3 (10.3)	0.4
Rhythm disorder	3 (15.8)	3 (10.3)	0.4
Other	2 (10.5)	6 (20.7)	0.5
No-flow time (minutes), median (IQR)	0 (0–0)	0 (0–3)	<0.01
Low-flow time (minutes), median (IQR)	40 (31–53)	60 (45–80)	0.02
Cardiac massage providers, *n* (%)			0.6
Non-HCP	7 (36.8)	10 (34.5)	1
Paramedics	4 (21.1)	11 (37.9)	0.3
Nurse	2 (10.5)	2 (7)	1
Doctor	6 (31.6)	6 (20.7)	0.5
Place of ECMO implantation, *n* (%)			0.2
Operating room	3 (15.8)	7 (24.1)	0.7
Cath Lab	7 (36.8)	9 (31)	0.8
Emergency room	9 (47.4)	8 (27.6)	0.2
ICU	0	5 (17.2)	0.1
EtCO_2_(mmHg), median (IQR)	25 (22–35)	25 (13–37)	0.9
pH before ECMO, median (IQR)	7.1 (6.9–7.2)	7 (6.9–7.1)	0.09
Lactate (mmol/L) before ECMO, median (IQR)	8.6 (6.7–12.1)	13.9 (10.8–15)	<0.01
Time on ECMO (hours), median (IQR)	96 (48–288)	24 (20–96)	<0.01
Age (years), median (IQR)	55 (43–62)	52 (48–60)	0.8
Gender, male, *n* (%)	11 (57.9)	23 (79.3)	0.2
Cardiovascular risk factor overall, *n* (%)	11 (57.9)	26 (89.7)	0.01
Overweight, *n* (%)	9 (47.4)	18 (62.1)	0.4
Hypertension, *n* (%)	4 (21.1)	13 (44.8)	0.08
Hypercholesterolemia, *n* (%)	7 (36.8)	10 (34.5)	0.6
Diabetes, *n* (%)	2 (10.5)	8 (27.6)	0.3
Smoker, *n* (%)	3 (15.8)	12 (41.4)	0.06

PEA: pulseless activity, VF: ventricular fibrillation, VT: ventricular tachycardia, ECMO: extracorporeal membrane oxygenation, EtCO_2_: end-tidal carbon dioxide, ICU: intensive care unit.

## Data Availability

The data presented in the manuscript are available from the corresponding author upon reasonable request.
